# Colocolic intussusception caused by a descending colon lipoma in an adult: a rare case report

**DOI:** 10.1097/RC9.0000000000000370

**Published:** 2026-03-10

**Authors:** Abhishek Acharya, Abhaya Acharya, Uttam Chaulagain, Kapil Khanal, Sushant Shah

**Affiliations:** a Maharajgunj Medical Campus, Institute of Medicine, Tribhuvan University, Kathmandu, Nepal; b Department of General Surgery, Tribhuvan University Teaching Hospital, Kathmandu, Nepal

**Keywords:** adult intussusception, bowel obstruction, colocolic intussusception, descending colon lipoma, submucosal lipoma

## Abstract

**Introduction:**

Adult intussusception is an uncommon clinical condition, accounting for only 5% of all intussusception cases and typically involving an identifiable pathological lead point, most often a neoplasm. Colocolic intussusception caused by a benign lipoma of the descending colon is exceptionally rare.

**Case presentation:**

A 47-year-old male presented with a 10-day history of intermittent periumbilical abdominal pain, loose stools, and fresh rectal bleeding. Imaging revealed a colocolic intussusception with a 5 × 4 cm fat-density lesion in the descending colon, suggestive of a submucosal lipoma. Exploratory laparotomy confirmed transverse-to-descending colonic intussusception with a lipoma serving as the lead point. Left hemicolectomy with side-to-side colocolic anastomosis was performed. Histopathology confirmed a benign submucosal lipoma.

**Discussion:**

Unlike pediatric cases, adult intussusception is usually secondary to an organic lesion. Lipomas are rare benign tumors, and those in the descending colon causing colocolic intussusception are especially unusual. Diagnosis often requires imaging, with CT scans being the gold standard. Surgical resection is the definitive treatment due to the high likelihood of pathology and risk of complications.

**Key clinical message:**

Colocolic intussusception due to descending colon lipoma is a rare but important differential in adults presenting with nonspecific abdominal symptoms and rectal bleeding. Timely imaging and surgical intervention are crucial for optimal outcomes.

**Conclusion:**

This case highlights the importance of maintaining high clinical suspicion and utilizing imaging in adults with vague gastrointestinal symptoms. Prompt surgical management of lipoma-induced intussusception can prevent severe complications.

## Introduction

Intussusception occurs when a segment of the bowel telescopes into an adjacent segment, resulting in obstruction and potentially leading to intestinal ischemia. This can cause several complications, including bowel obstruction, tissue death (necrosis), and sepsis. While it is more frequently seen in children, it is rare in adults. However, when it does occur in adults, it is often associated with an underlying pathological lead point, such as a neoplasm^[^1,2^]^.

Diagnosing intussusception in adults is challenging and requires a high index of clinical suspicion. This difficulty arises because abdominal pain – a common and often nonspecific complaint in the emergency department – can have numerous causes. The evaluation and management of abdominal pain largely depend on the severity of the patient’s signs and symptoms. While history, physical examination, and laboratory tests can provide valuable clues, imaging is typically necessary to confirm the diagnosis. The condition is further complicated by the fact that intussusception in adults often resembles many other possible diagnoses^[^3^]^.

If not accurately diagnosed, intussusception can result in serious complications that may negatively impact patient outcomes. Surgical intervention is the definitive treatment, and achieving favorable outcomes relies on prompt diagnosis and the coordinated efforts of an interprofessional team, including physicians, nurses, and technicians. This discussion emphasizes a comprehensive understanding of this rare but potentially life-threatening condition^[^1^]^.

Adult intussusception represents 1% of all bowel obstructions, 5% of all intussusceptions, and 0.003%–0.02% of all hospital admissions^[^4^]^. Unlike in children, in adults, nearly 90% of cases have a pathological lead point, most commonly being neoplasm^[^5^]^. Other risk factors include mass (benign or malignant), anatomical changes, post-surgical adhesion, endometriosis, idiopathic, fibroids, gastrostomy tube, and jejunostomy tube^[^1,6^]^.

This case report was prepared in accordance with SCARE guidelines 2025^[^7^]^.

## History

A 47-year-old male presented to the surgical outpatient department at tertiary center with a 10-day history of periumbilical abdominal pain and altered bowel habits. The pain was colicky, mild to moderate in intensity, intermittent, and localized to the periumbilical region. He reported passing loose stools seven times daily, initially mixed with mucus, followed by the appearance of fresh, bright red blood per rectum after a few days. Associated symptoms included nausea, occasional non-bilious vomiting, and mild abdominal distension. There was no history of fever, anorexia, or significant weight loss. The patient denied any prior similar episodes, previous abdominal surgeries, or known comorbidities.

## Examination

On physical examination, the patient appeared clinically stable and in fair general condition. Vital signs were within normal limits. Abdominal examination revealed a soft, non-distended abdomen without tenderness, palpable masses, or signs of peritonism. Bowel sounds were normal and audible. Hernial orifices were free. Rectal examination showed an empty rectal ampulla.

### Investigations

**Figure 1.**
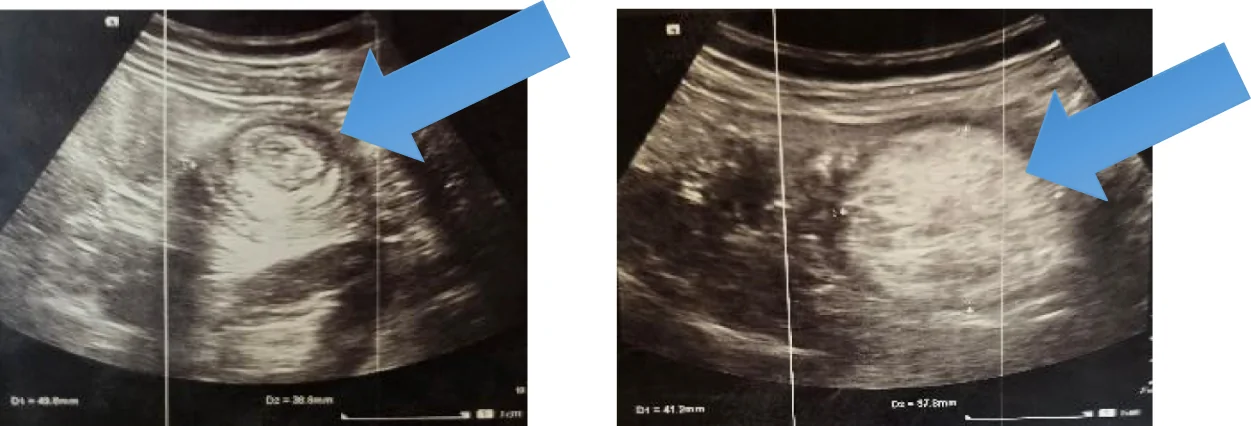
Abdominal ultrasound: Target-like lesion measuring 49 × 38.8 mm in left upper quadrant; pseudokidney sign on longitudinal view; bright lesion 4.1 × 3.7 cm inside bowel just beyond intussusception.

**Figure 2.**
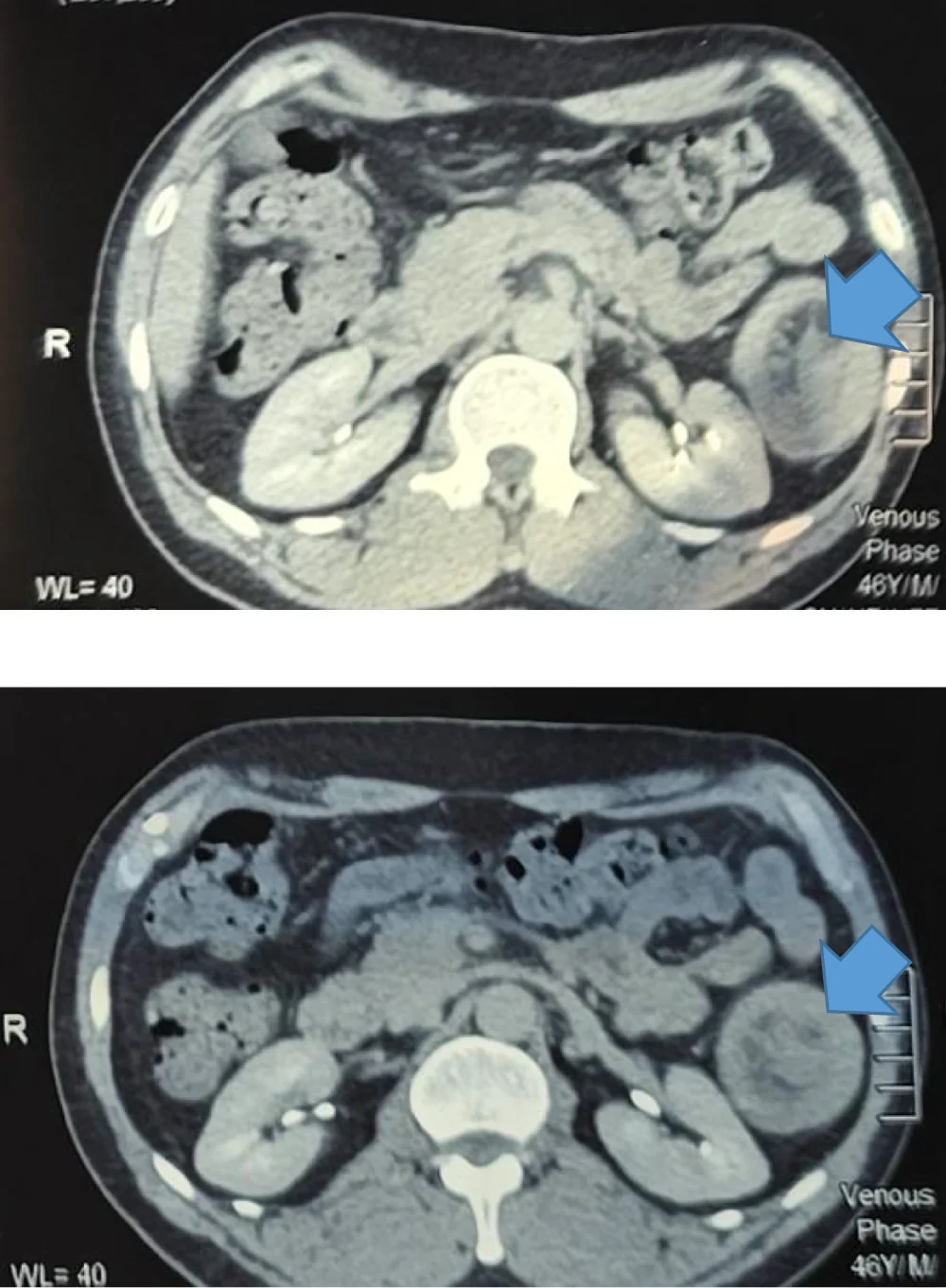
CT-scan: Transverse colon telescoping into splenic flexure and descending colon; fat-density mass measuring approximately 5 × 4 cm in descending colon; no bowel ischemia or perforation.

Laboratory tests showed mild anemia with a hemoglobin level of 11.8 g/dL, a normal white blood cell count of 8100 cells/mm^3^, and a mildly elevated C-reactive protein level of 31.88 mg/L. Stool examination revealed red blood cells and mucus but was negative for parasites or infectious organisms. Abdominal ultrasound demonstrated a target-like lesion measuring about 49 × 38.8 mm in the left upper quadrant with a pseudokidney sign on the longitudinal view. Just beyond this intussusception, a well-defined bright lesion measuring 4.1 × 3.7 cm was seen inside the bowel (Fig. 1). A CT scan confirmed the diagnosis of colocolic intussusception, showing the transverse colon telescoping into the splenic flexure and descending colon (Fig. 2). A clearly outlined, fat-density mass about 5 × 4 cm was identified in the descending colon, consistent with a submucosal lipoma. There was no evidence of bowel ischemia or perforation (Table 1).

**Table 1:** Summary of laboratory and radiological findings in a patient with colocolic intussusception secondary to a descending colon lipoma.

Investigation	Findings
Hemoglobin	11.8 g/dL
White blood cell count	8100 cells/mm^3^
C-reactive protein (CRP)	31.88 mg/L
Stool examination	Red blood cells and mucus present; no parasites or infectious organisms detected
Abdominal ultrasound	Target-like lesion measuring 49 × 38.8 mm in left upper quadrant; pseudokidney sign on longitudinal view; bright lesion 4.1 × 3.7 cm inside bowel just beyond intussusception
CT scan	Transverse colon telescoping into splenic flexure and descending colon; fat-density mass measuring approximately 5 × 4 cm in descending colon; no bowel ischemia or perforation

### Differential diagnosis

The clinical presentation and imaging strongly suggested colocolic intussusception caused by a benign colonic lipoma. Other possible diagnoses included malignant tumors such as adenocarcinoma, large adenomatous polyps, or inflammatory bowel disease. However, the lack of systemic symptoms, the characteristic fat density seen on imaging, and absence of surrounding inflammation made these alternatives less likely.

HIGHLIGHTS

- Adult colocolic intussusception due to a descending colon lipoma is an exceptionally rare clinical entity.
- The patient presented with nonspecific gastrointestinal symptoms including abdominal pain, loose stools, and rectal bleeding.
- Preoperative CT imaging was instrumental in identifying both the intussusception and the underlying lipoma.
- A large submucosal lipoma (5 × 4 cm) served as the pathological lead point for the intussusception.
- Open surgical resection (left hemicolectomy with colocolic anastomosis) was performed to manage the condition.
- Histopathological examination confirmed a benign submucosal lipoma without evidence of malignancy.
- This case emphasizes the importance of early diagnostic imaging and timely surgical intervention in adult intussusception.
- The report contributes to the limited literature on descending colonic lipomas causing colocolic intussusception in adults.

### Management

**Figure 3.**
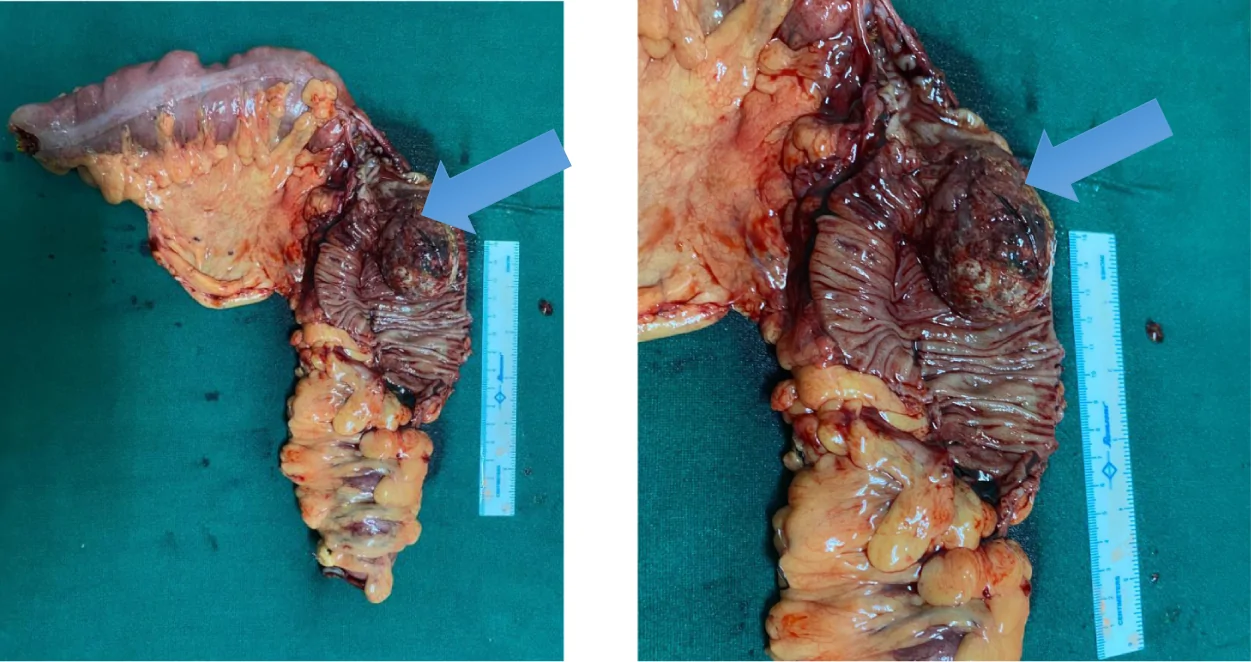
Gross specimen of the resected colon showing a well-defined submucosal lipoma serving as the lead point for colocolic intussusception. The lesion appears polypoid with overlying mucosal congestion and ulceration.

**Figure 4.**
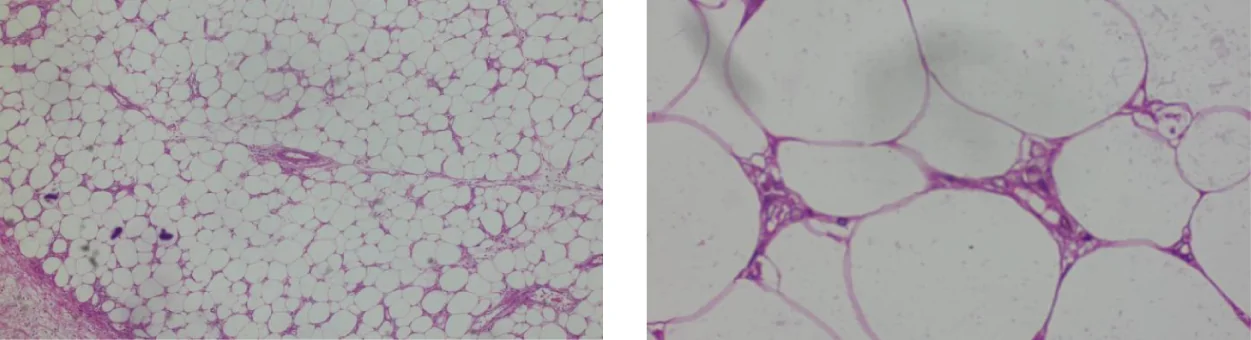
Histopathological examination showing a benign submucosal lipoma without dysplasia or malignancy.

After appropriate preparation, an exploratory laparotomy was performed. Intraoperatively, the transverse colon was found telescoped into the splenic flexure and descending colon, with a 5 × 4 cm polypoid lipoma serving as the lead point. The splenic flexure and descending colon were mobilized, preserving the left colic and middle colic arteries to maintain blood supply. Resection was performed from the distal transverse colon through the descending colon to the sigmoid colon. On-table colonic lavage was performed using warm normal saline (approximately 6–8 L) via a Foley catheter inserted through a proximal enterotomy in the transverse colon, with effluent drained through the open distal bowel until clear, prior to construction of the anastomosis. A side-to-side colocolic anastomosis was created using an 80 mm triple stapler and reinforced with 3-0 Prolene sutures; the anastomosis was confirmed to be patent and tension free. A 28 French drain was placed in the left pelvic cavity. Histopathology confirmed a benign submucosal lipoma without dysplasia or malignancy (Figs. 3 and 4). The patient had an uneventful postoperative recovery and was discharged on postoperative day 6 in stable condition. At the 2-week follow-up, the surgical wound had healed well, and the patient reported resolution of abdominal pain and normalization of bowel habits. He was able to resume a regular diet and daily activities without discomfort. Long-term surveillance, including colonoscopic examination, was recommended to screen for any additional colonic lesions and ensure ongoing gastrointestinal health.

## Discussion

Intussusception in adults is a rare clinical entity, distinctly different from the more common pediatric counterpart in terms of etiology, presentation, and management. While pediatric cases are predominantly idiopathic, adult intussusception is typically secondary to an identifiable pathological lead point^[^4^]^. Among these, benign adipose tissue tumors, primarily lipomas, represent an uncommon but significant cause^[^8^]^.

In this case, a 47-year-old male presented with classical yet nonspecific symptoms, and imaging revealed a colocolic intussusception involving the transverse and descending colon, secondary to a large submucosal lipoma. This case illustrates an uncommon anatomical pattern and etiology, reinforcing the need for careful diagnostic evaluation in adults with vague abdominal complaints. The pathophysiology of intussusception involves the invagination of one segment of the bowel (intussusceptum) into an adjacent segment (intussuscipiens), typically due to an intraluminal or mural lesion that alters the peristaltic wave and traction forces^[^9^]^.

In adults, over 90% of intussusceptions have a structural cause, with benign tumors, malignant neoplasms, inflammatory lesions, and iatrogenic factors being potential culprits^[^10^]^. Submucosal lipomas, especially those greater than 2 cm, can serve as effective lead points due to their size, pedunculated shape, and intraluminal protrusion. Their soft consistency allows progressive telescoping of the bowel segment during peristalsis^[^11^]^. Clinically, adult patients present with chronic or intermittent symptoms including abdominal pain, nausea, vomiting, altered bowel habits, and in some cases, rectal bleeding^[^10^]^.

In our case, the patient’s symptoms – intermittent colicky periumbilical pain, loose stools, and fresh per rectal bleeding – were consistent with intussusception but lacked specificity, mirroring the diagnostic challenge frequently encountered in such presentations. Adult intussusception is rare, accounting for approximately 5% of all intussusception cases and 1%–5% of adult bowel obstructions^[^12^]^. Among adult intussusceptions, colocolic types are less common than enteric ones. Colonic lipomas are infrequent benign tumors, with an incidence between 0.2% and 4.4% of colonic tumors. Over 70% occur in the right hemicolon, making descending colon involvement uncommon^[^13^]^. Even fewer cases have been reported where a descending-colon lipoma causes a colocolic intussusception extending from the transverse colon into the descending colon. The review by Tasselli *et al* showed that while 28% of lipoma-induced colocolic intussusceptions occur in the transverse colon, only about 14% involve the descending colon^[^14^]^.

One of the few dedicated reports comes from Wang *et al* (1992), describing an adult case of descending-colon lipoma leading to colocolic intussusception^[^15^]^. This makes this case both anatomically and etiologically uncommon. Given the vague nature of symptoms in adult intussusception, imaging plays a central role in diagnosis. Ultrasound can offer crucial early clues, as in this case where a “target sign” on transverse imaging and a “pseudokidney sign” on longitudinal view strongly suggested intussusception^[^16^]^.

A hyperechoic, well-defined lesion distal to the intussuscepted segment hinted at a possible lead point. CT imaging remains the gold standard for diagnosis. It not only confirms the presence of intussusception but also characterizes the nature of the lead point^[^12^]^. In this case, the CT scan demonstrated classic features of colocolic intussusception and identified a 5 × 4 cm fat-density lesion with sharp margins, favoring a lipoma. Importantly, there were no signs of ischemia, perforation, or regional lymphadenopathy, which reduced the suspicion of malignancy and guided operative strategy. Routine laboratory investigations revealed mild anemia and raised C-reactive protein levels, supporting an inflammatory or bleeding process. Stool analysis demonstrated red blood cells and mucus but no infectious agents, further corroborating a mechanical rather than infectious etiology.

Management of adult intussusception differs fundamentally from pediatric cases. Given the high rate of underlying pathology, particularly neoplastic lesions, surgical resection is typically advocated rather than conservative management used in children^[^10^]^. The choice between reduction before resection or primary resection without reduction depends on several intraoperative and preoperative factors such as the lesion’s location, clinical suspicion for malignancy, bowel viability, and presence of complications like ischemia or perforation^[^17^]^. In our patient, the presence of a sizable (5 × 4 cm) submucosal lipoma as the lead point, coupled with the risk of recurrence and potential complications associated with simple reduction, justified performing a left hemicolectomy with primary colocolic anastomosis. Importantly, our surgical technique emphasized meticulous preservation of the left colic artery and the left branch of the middle colic artery to maintain adequate vascular perfusion, a step that may decrease anastomotic complications and is not uniformly highlighted in the literature^[^18^]^. The resection margins extended from the distal transverse colon to the sigmoid colon, delineated by a clear intraoperative demarcation line, potentially reducing the risk of residual disease or recurrence compared to more conservative resections^[^19^]^.

Additionally, intraoperative colonic lavage was performed to reduce bacterial load and fecal contamination prior to reconstruction, an approach supported by evidence to minimize postoperative infectious complications^[^20^]^. Our use of a stapled side-to-side anastomosis reinforced with 3-0 Prolene sutures ensured a secure and tension-free anastomosis, which may improve healing and reduce leakage rates compared to unstapled or unreinforced techniques^[^21^]^. Placement of a pelvic drain facilitated early detection and management of possible postoperative complications. Collectively, these surgical refinements underscore a comprehensive and cautious operative strategy aimed at optimizing patient outcomes.

Multiple case reports and small series have documented adult intussusception caused by colonic lipomas, but the majority occurs in the right colon. Adult colonic intussusception caused by lipomas has been described in numerous case reports and small series, with the majority of cases occurring in the right colon, particularly the cecum and ascending segments^[^17^]^. Right-sided involvement predominates, while the descending colon accounts for a minority of cases – approximately 13%–15%^[^10^]^. Left-sided intussusceptions, especially those involving the descending colon, are considerably less frequent. Even rarer is the anatomical subtype involving transverse-to-descending colonic intussusception, which has been reported in only a handful of adult cases worldwide^[^22^]^. This highlights the exceptional rarity of such presentations and underscores the importance of maintaining a high index of suspicion in atypical clinical scenarios. While most documented cases report similar clinical presentations, the surgical approach varies significantly. Some authors have favored laparoscopic resections or en bloc removal, particularly in cases where malignancy could not be definitively excluded preoperatively^[^23^]^.

Our case is consistent with this surgical philosophy and adds to the scarce literature highlighting the descending colon as a potential origin site for intussusception. It supports the role of modern imaging in preoperative diagnosis and strengthens the recommendation for surgical resection in adults with structural lead points.

This case report is limited by the absence of colonoscopic evaluation prior to surgery, which may have offered visual and histologic confirmation of the lesion. However, due to the acute symptomatic presentation and supportive imaging findings, surgical exploration was appropriately prioritized. Additionally, long-term follow-up data are not yet available, and advanced imaging or immunohistochemical staining of the tumor was not performed, which could provide further insight into tumor biology. Being a single case, its generalizability is limited but nonetheless contributes to rare-case awareness.

## Conclusion

Colocolic intussusception in adults is a rare but important clinical entity, often overlooked due to its nonspecific presentation. When caused by a benign lesion like a lipoma, timely diagnosis and management can prevent severe complications. This case reinforces the need for high clinical suspicion, the value of imaging in diagnosis, and the central role of surgery in treatment. Clinicians should consider lipomas in the differential diagnosis of adult patients with intermittent abdominal pain and rectal bleeding. A multidisciplinary approach ensures timely diagnosis, optimal management, and prevention of complications such as obstruction, ischemia, or perforation.

## Data Availability

The data that support the findings of this study are available from the corresponding author upon reasonable request.
